# On the nature of the interlayer magnetic interactions in biphase ferromagnetic films

**DOI:** 10.1038/s41598-023-31563-2

**Published:** 2023-03-17

**Authors:** E. F. Silva, M. Gamino, A. B. Oliveira, M. A. Correa, F. Bohn

**Affiliations:** Departamento de Física, Universidade Federal do Rio Grande do Norte, 59078-900 Natal, RN Brazil

**Keywords:** Magnetic properties and materials, Sensors and biosensors

## Abstract

We report on the nature of the interlayer magnetic interactions in NiFe/Cu/Co films. By probing the quasi-static and dynamic magnetic properties of biphase ferromagnetic films, with soft and hard ferromagnetic phases intermediated by a non-magnetic layer, we address aspects of the coupling between magnetic layers. Our results demonstrate the nature of the interlayer magnetic coupling in biphase films. We also disclose the asymmetric magnetoimpedance effect as a fingerprint of the nature of the magnetic interlayer interactions playing key role in the magnetization dynamics of the system. We revisit in literature data and ideas on the asymmetric magnetoimpedance and the nature of the magnetic interactions in biphase ferromagnetic systems. Then, we compare our findings with results for biphase ribbons and microwires. Our observations raise the fundamental similarities and differences in the asymmetric magnetoimpedance of these structures.

## Introduction

Bilayers, trilayers, spin valves, pseudo-spin valves, i.e. multilayers, are nanostructured systems whose magnetic response is dependent on properties of the individual ferromagnetic (FM) layers, and on how such layers interact. In the presence of a non-magnetic (NM) layer intermediating the magnetic ones, the nature of the interaction between magnets is settled according to the sample dimensions, spacer thickness, material of the NM layer and features of the interfaces^1–4^.

In FM/NM/FM multilayers, the effective interlayer magnetic coupling is a result of the competition between contributions coming from four different origins: the direct exchange coupling through pinholes in the spacer^5^; the indirect exchange coupling through Ruderman-Kittel-Kasuya-Yosida (RKKY) interactions^6–8^; the long-range dipolar coupling due to correlated roughness at both spacer interfaces, a.k.a. Néel (orange-peel) coupling^9–11^; and, finally, the so-called magnetostatic coupling, a long-range dipolar coupling as well, but that takes place through stray fields^12–14^. These interactions are among the key ingredients determining the magnetic response of the system. Therefore, they are of utmost importance to the understanding of magnetic nanostructures, from fundamental physics perspective and for technological applications.

Manifestations ascribed to the interlayer magnetic interactions have been reported in the last years through different ways, ranging from techniques probing the quasi-static magnetic properties, as the familiar magnetometry^14–16^ and the magnetoresistance^17–19^, till the dynamic magnetic response, considering for instance the effects of ferromagnetic resonance^20–22^ and magnetoimpedance^23,24^. Of our concern, here we concentrate on the magnetoimpedance effect (MI). MI consists in the change of the complex electrical impedance of a magnetic material submitted to a magnetic field, and brings information on the magnetization dynamics over a wide range of fields and frequencies^25^. The effect is controlled by the transverse magnetic permeability, which in turn depends on structural and magnetic parameters, as effective anisotropy, magnetic damping, and geometry and thickness of the sample^26^. Hence, it provides insights on magnetic anisotropy, uniformity of the magnetization, energy terms affecting the magnetic behavior, and the nature of interactions governing the magnetization dynamics^27,28^.

Since the discovery of the effect, MI has been extensively investigated in a variety of magnetic systems^27,28^. Soft magnetic materials are highly sensitive to small field variations at low magnetic fields, but most of them have essentially nonlinear MI behavior around zero magnetic field, which prevents a simple straightforward derivation of an appropriate signal for sensor applications^29^. Although much effort has been made to the exploration of magnetic materials with specific features for application as sensor elements in devices, MI has yet been taken as a playground for investigations on the physics behind the effect and the mechanisms controlling the dynamic magnetic response. More recently, it has been shown that materials exhibiting asymmetric magnetoimpedance (AMI) effect, such as wires^29–31^, ribbons^23,24,32–34^, and films^35–41^, arise as promising candidates for applications, opening possibilities for the use of this kind of materials in auto-biased linear magnetic field sensors. Remarkably, for biphase ferromagnetic systems, with hard and soft ferromagnetic phases intermediated by a non-magnetic layer acting together, AMI has been linked with the existence of interactions between magnetic layers^27,28^. It is the central topic we address here.

In this article, we report on the nature of the interlayer magnetic interactions in biphase ferromagnetic NiFe/Cu/Co films. After nearly 20 years of studies on MI, we understand the need of reviving data and ideas, and raise the fundamental similarities and differences in the asymmetric magnetoimpedance in different biphase ferromagnetic systems. Thus, we make a tour through the asymmetric magnetoimpedance and the nature of the magnetic interactions by revisiting results found in literature for ribbons and microwires, and compare them with our findings for films. Our observations raise the fundamental similarities and differences in the magnetoimpedance response of these structures and unravel the nature of interlayer magnetic interactions in biphase ferromagnetic films.

## Results

### Scrutinizing biphase ferromagnetic films

Here, we focus on NiFe/Cu/Co films as a playground for investigating fundamental aspects of the magnetic interactions in biphase ferromagnetic nanostructures. In this work, we reproduce Ni$_{81}$Fe$_{19}$(25 nm)/Cu($t_{\mathrm {Cu}}$)/Co(50 nm) films, with $t_{\mathrm {Cu}}= 5$, 7, and 10 nm (see “Methods” section for details on the production of the samples and magnetic characterization), previously studied in Ref.^39^. These thicknesses of the non-magnetic Cu layer were particularly chosen to produce biphase films exhibiting AMI^39^. Besides checking the response of the magnetoimpedance effect in such nanostructures, we explore the magnetization reversal through magnetization and magnetoresistance curves. Thereby, by careful experiments of minor magnetization loops, we are then able to identify the nature of the interlayer magnetic coupling in biphase ferromagnetic films.

### Asymmetric magnetoimpedance effect

**Figure 1:**
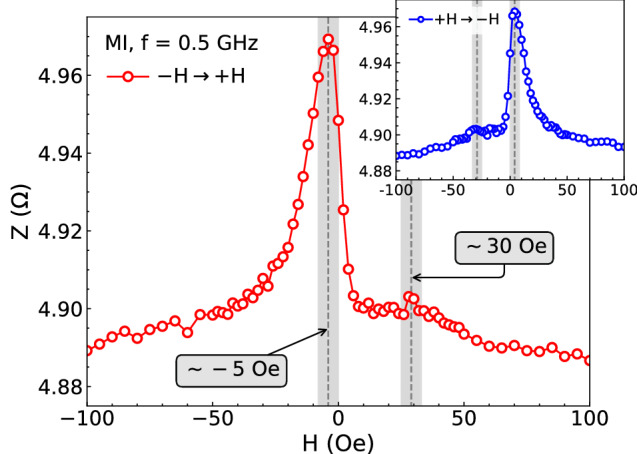
The MI curve for increasing field at the frequency of 0.5 GHz for our NiFe/Cu/Co film with $t_{\mathrm {Cu}} = 10$ nm. The gray zones indicate the position in field of the MI peaks. Specifically, for this film with $t_{\mathrm {Cu}} = 10$ nm, they are found at $\sim -\,5$ and $\sim 30$ Oe. The MI curve taken when the field goes from positive to negative values is shown in the inset. In this case, the reverted asymmetric behavior is verified; and the gray zones locate the position in field of the MI peaks as well, here at $\sim 5$ and $\sim -\, 30$ Oe.

As starting point for our analysis, we address the results of magnetoimpedance effect for ferromagnetic NiFe/Cu/Co films, having Cu thickness varying from 0 to 10 nm, uncovered in Ref.^39^. From such report, we observe an evolution in the shape of the magnetization curves and MI effect with the thickness of the Cu spacer $t_{\mathrm {Cu}}$ (as we can see in Figs. 1 and 2 of Ref.^39^, respectively), indicating the existence of a spacer thickness range close to $\sim 3$ nm splitting the films in two groups according to the magnetic response.

First, films with $t_{\mathrm {Cu}}$ below 3 nm present the well-known, ordinary MI behavior for anisotropic systems. It is characterized by the double peak behavior, symmetrical at around $H=0$, with the peaks having roughly similar amplitude. At low and intermediate frequencies, the positions of the peaks remain unchanged close to the effective anisotropy field of the sample; and the peaks displace towards higher fields as the frequency increases (see Fig. 3 of Ref.^39^). At the same time, the angular dependence of the magnetization curves indicates an effective uniaxial in-plane magnetic anisotropy (Fig. 1 of Ref.^39^). Such magnetic response is an indicative that the NiFe and Co layers are ferromagnetically coupled, interacting via direct exchange coupling through pinholes in the non-magnetic spacer or between touching ferromagnetic layers, so that they behave like a single one.

More interestingly, for $t_{\mathrm {Cu}}$ above $\sim \,\,3$ nm, the films exhibit a biphase magnetic behavior and AMI. In Fig. 1, we present a representative result of the AMI for our biphase ferromagnetic NiFe/Cu/Co films reproduced for this work. Although the curves are acquired over a complete magnetization loop (the inset in Fig. 1 brings to light the MI result for decreasing field, revealing the reverted AMI behavior), here we focus our analysis in just part of the curve, when the field goes from negative to positive values﻿. The asymmetric behavior is assigned by two features: shift in field of the MI curve, depicted by the asymmetric position of the peaks, and asymmetry in shape, evidenced by the difference of amplitude of the peaks. Generally, between 0.1 and $\sim 1.5$ GHz, where the skin effect commands the dynamical behavior, the MI curves are asymmetric with a linear transition region at low fields, and the positions of the peaks remain invariable for each sample. Remarkably, the peaks are located at distinct field values in the negative and positive ranges, as we can confirm from the gray zones highlighted in Fig. 1. With increasing frequency (not shown here), above $\sim 1.5$ GHz, as a clear signature of the strong skin and FMR effects as responsible by the MI variations, we verify the displacement of the peaks towards higher fields, suppressing the asymmetry of the peaks, in position and amplitude, resulting in symmetric peaks around $H=0$ with same amplitude (such dependence with frequency may be clearly visualized in Fig. 3 of Ref.^39^). It is worth highlighting that similar AMI behavior is also found in biphase ferromagnetic films with distinct non-magnetic metallic spacers^40^, as well as multilayered biphase films^41^. Despite the differences in the nature of the materials and the number of the base elements, all these nanostructures share the main ingredients influencing the magnetic response.

In ferromagnetic materials, AMI may occur naturally at small dc-bias magnetic fields below the anisotropy field, due to the application of a dc-bias current, an ac-bias field or exchange bias^28^. The AMI response in multilayers in turn is often understood as a signature of the existence of interactions between magnetic layers. The question then arises is what interlayer magnetic interaction plays the major role in the energy balance affecting the transverse magnetic permeability of the system.

### Magnetization reversal in biphase ferromagnetic films

**Figure 2:**
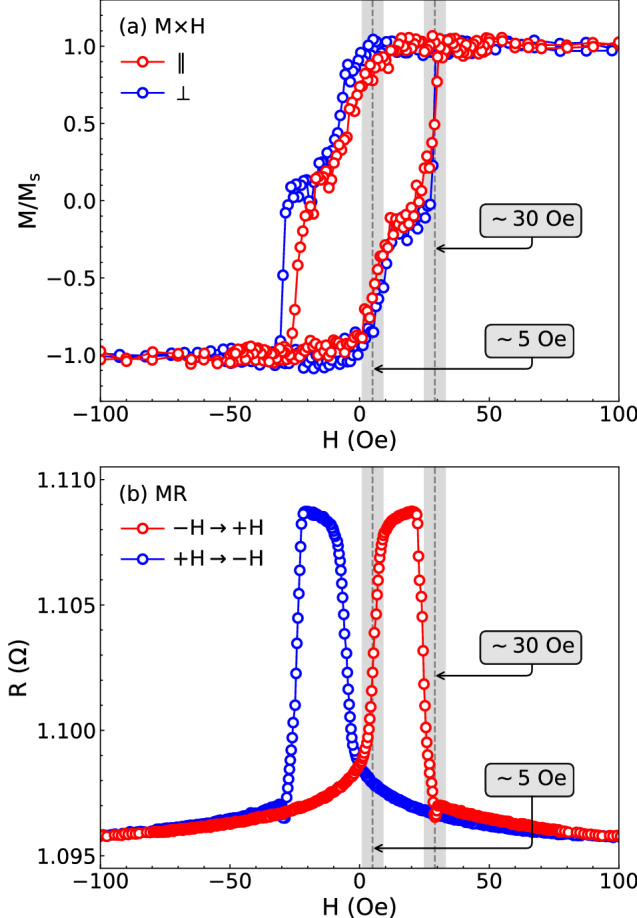
(**a**) Normalized magnetization and (**b**) magnetoresistance curves for our NiFe/Cu/Co film with $t_{\mathrm {Cu}} = 10$ nm. The magnetization curves are measured with in-plane magnetic field applied along (red) and perpendicular (blue) to the main axis of the sample. The magnetoresistance curve is acquired with the electrical current applied along the main axis, and the color code represents the responses obtained in the two branches of the hysteresis loop, with increasing (red) and decreasing (blue) fields. The gray zones indicate the regions of magnetization reversion of the NiFe layer at low field and of the Co one at higher field in the branch of the hysteresis loop with increasing field from the maximum negative value. Specifically, for the film with $t_{\mathrm {Cu}} = 10$ nm, they are found at $\sim 5$ and $\sim 30$ Oe.

As a first action to identify the type of interaction in our biphase films, we look at their magnetization and magnetoresistance curves. Figure 2 displays the results for our film with $t_{\mathrm {Cu}}$ of 10 nm. From the magnetization in Fig. 2a, we verify that the curves taken along and perpendicular to the main axis have quite-similar features. They are assigned by a two-stage magnetization process, which is characterized by the magnetization reversion of the soft NiFe layer at low field, followed by the reversion of the hard Co layer at higher field. In principle, the biphase magnetic behavior suggests that the ferromagnetic layers are uncoupled, so such magnetization curves do not provide us any clear fingerprint of the existence and/or type of interaction between magnetic layers.

Regarding the magnetoresistance in Fig. 2b, it brings insights on the orientations between the magnetizations of the ferromagnetic layers at different magnetic field values, as well as on the magnetization reversal process previously discussed. The largest resistance values are found when an antiparallel alignment between the magnetizations of the soft and hard ferromagnetic layers is established, while the smaller ones occur in the case of the parallel alignment^17,42–44^. Thus, when the field is swept from the maximum negative to the positive values, the abrupt rise of the resistance occurs at the switching field of the soft NiFe layer, where the magnetic configuration passes from a parallel to an antiparallel alignment between the ferromagnetic layers. As the field continuously increases, the sudden drop in the resistance is located at the switching field of the hard Co layer. Similar behavior is verified in the magnetoresistance curve along the branch of the hysteresis loop with decreasing field. Given the stated above, the magnetoresistance curves corroborate the magnetization reversion fields identified from magnetometry, highlighted by the gray zones in Fig. 2, but they do not carry any specific signature of interlayer interactions too.

### Revealing the interlayer magnetic coupling in biphase films

Going beyond, in order to check the existence and the nature of the coupling between ferromagnetic soft NiFe and hard Co layers, we obtain minor magnetization loops for our films. This measure consists of pre-magnetizing the entire biphase film, and, subsequently acquiring the magnetization in a restricted low-field interval, in which only the reversion of soft magnetic layer is allowed, whilst the magnetization of the hard one remains unchanged, in its saturated state.

**Figure 3:**
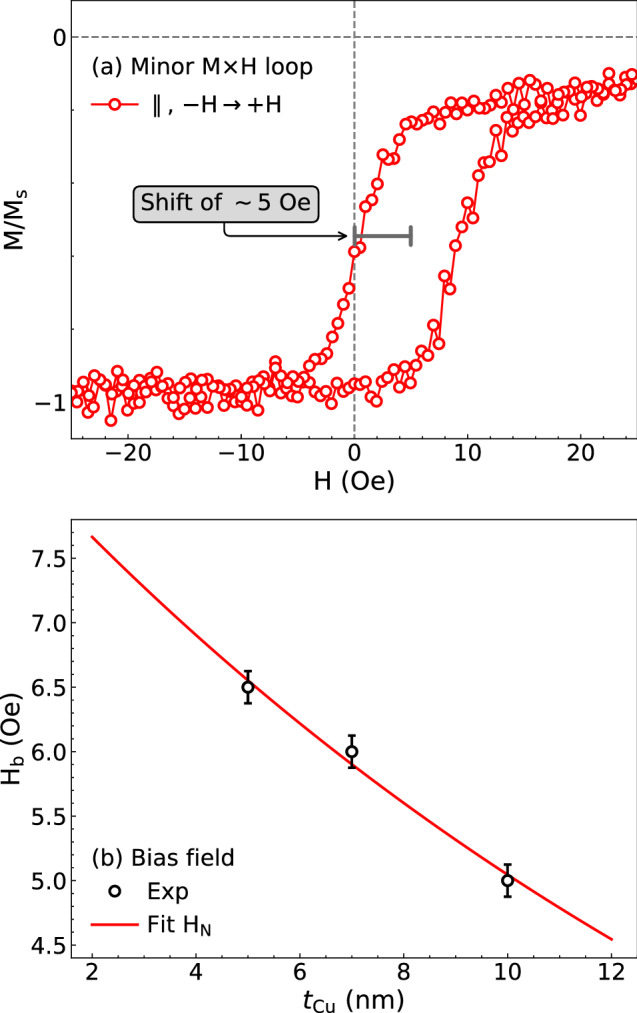
(**a**) Minor magnetization loop for our Nife/Cu/Co film with $t_{\mathrm {Cu}} = 10$ nm. The curve is measured with the in-plane magnetic field applied along the main axis of the sample, starting from the negative pre-magnetizating field of $-300$ up to 25 Oe. Specifically, for such film, the bias field is $H_{\mathrm {b}} \sim 5$ Oe. (**b**) Bias field $H_{\mathrm {b}}$ as a function of $t_{\mathrm {Cu}}$ obtained from the minor magnetization loops for our biphase films. The solid line is a fit obtained using Eq. (1). By varying $t_{\text {NM}} = t_{\text {Cu}}$ and assuming $M_{\text {s}} = M_{\text {s, NiFe}} = 780$ emu/cm$^3$ and $t_{\text {FM}} = t_{\text {NiFe}} = 25$ nm, from the fit we find $h=2.58 \pm 0.09$ nm and $\lambda =170 \pm 19$ nm, which fall into the range of values often found for similar structures^13,45,46^.

Figure 3a shows the minor magnetization loop for our biphase film with $t_{\mathrm {Cu}}$ of 10 nm. Here we achieve the minor loop starting from a negative field with amplitude to saturate the sample, leading to the parallel alignment of both NiFe and Co ferromagnetic layers. Then, the applied field is reduced to zero and reversed up to the maximum positive value of 25 Oe, following back to the maximum negative field. The existence of an interlayer interaction may be identified through the shift in field of the magnetization curve with respect to $H=0$. Such shift is ascribed to the presence of an effective bias field $H_{\mathrm {b}}$ acting on the soft ferromagnetic layer. In other words, there is a bias field originated from the hard Co layer that induces an unidirectional magnetic anisotropy in the soft NiFe layer, hence shifting the minor magnetization curve — in a similar way to the exchange bias effect found for films with ferromagnetic/antiferromagnetic layers with exchange coupling.

Based on the shift of the minor magnetization loop, the origin of the interaction may be set; the shift of the curve is dependent on the orientation of the pre-magnetizing field, and we may infer an antiferromagnetic-like coupling when pre-magnetizing and bias fields have the very same signal, otherwise the coupling is ferromagnetic^14,15,47^. In our case, for the *H*-sweep from $-H$ to $+H$, the coupling is ferromagnetic for $H_{\text {b}} > 0$ and antiferromagnetic for $H_{\text {b}} < 0$. Here, we achieve the minor loop employing a negative pre-magnetization field, and find a displacement of the curve to positive field values. It can be understood as a signature of an interlayer interaction of ferromagnetic-like nature, favouring the parallel alignment of the soft NiFe and hard Co layers.

We verify positive $H_{\text {b}}$ for all of our films, as depicted in Fig. 3b. We can yet notice that $H_{\text {b}}$ gradually decreases with increasing $t_{\mathrm {Cu}}$, suggesting the coupling is dependent on the spacer thickness. It is known that both exchange coupling and dipolar interaction decrease with raising the thickness of the spacer^9,17,45^. In the case of our films, we understand that contributions of direct exchange coupling through pinholes in the spacer and of indirect exchange coupling through RKKY interactions may not be considered, due to the high thickness range of the spacer explored in our samples, $t_{\mathrm {Cu}} \ge 5$ nm. Hence, by exclusion, we demonstrate that dipolar interactions have the major role in the magnetic response of our biphase films.

Magnetostatic and Néel (orange-peel) couplings are long-range interactions of dipolar origin. The first arises in biphase magnetic systems separated by a non-magnetic layer as a result of the interaction between adjacent layers with different magnetic properties. Nevertheless, this interlayer magnetic interaction is proven for promoting an antiferromagnetic-like coupling^17,18^. So, through the evidences raised from the minor magnetization loops for our films, the magnetostatic coupling can be also set aside. Although the idea of a magnetostatic coupling may be abandoned, its existence in biphase ferromagnetic films can not be completely disregarded; whether magnetostatic coupling is still present despite the evidences, we can for sure infer that it has a minor contribution in the energy balance.

Finally, only the Néel (orange-peel) coupling remains. It is a long-range dipolar interaction between adjacent ferromagnetic layers due to the correlated waviness of the magnetic layers^9,10^. The effect of the Néel coupling can be understood, at least qualitatively, by considering the distribution of unbalanced “magnetic charges” near the ends of the hard magnetic layer, giving rise to a bias field induced by divergences of magnetization mainly due to roughness in the interfaces and limits of the sample^29,47^. The bias field penetrates the non-magnetic spacer and acts on the soft magnetic layer through a ferromagnetic-like coupling.

The Néel field may be written as^9^

1 $$\begin{aligned} H_N=\frac{\pi ^2}{\sqrt{2}}\left( \frac{h^2}{\lambda t_{{\mathrm {FM}}}}\right) M_{{\mathrm {s}}} \,\exp \left( \frac{-2\pi \sqrt{2}t_{{\mathrm {NM}}}}{\lambda }\right) , \end{aligned}$$

where *h* and $\lambda$ are the amplitude and wavelength of the roughness profile, $t_{{\mathrm {FM}}}$ and $t_{{\mathrm {NM}}}$ are the thickness of the soft magnetic layer and non-magnetic spacer, respectively, and $M_{{\mathrm {s}}}$ is the saturation magnetization of the soft magnetic layer.

To confirm the nature of the interlayer magnetic interaction in our biphase films, we probe the dependence of $H_{\mathrm {b}}$ taken from the minor magnetization loops and verify whether it is compatible with the behavior expected for the Néel field with $t_{\text {Cu}}$. Figure 3b brings to light the fit of the experimental data using Eq. (1). Notice the quite-good agreement between experiment and theory. Therefore, we corroborate that Eq. (1) is suitable for describing the behavior of the bias field with the spacer thickness.

Given all features found for our biphase NiFe/Cu/Co films, we interpret our results as unambiguous signatures of the Néel (orange-peel) coupling playing the key role in the magnetic response and magnetization dynamics of the system.

## Discussion

Here, at the same time we explore and discuss findings achieved for our biphase ferromagnetic samples, we make an excursion through results found in literature for biphase wires^29,30^, ribbons^32–34^, and films^39–41^. It is interesting to notice that the asymmetric magnetoimpedance response verified for biphase films as the ones explored here have similarities and differences when compared with results found for biphase ferromagnetic systems having dissimilar geometries. As similarities, we may raise the profile of the AMI curves, having asymmetric position of the peaks and difference of peaks amplitude. As fundamental distinction, the most striking finding resides in the relative position in field of the high and low-amplitude peaks according to the pre-magnetizing field employed in the experiment. Such feature is straightforwardly related with the nature of the interaction between ferromagnetic layers and the bias field acting on the soft layer^48,49^.

Picking up some results found in literature for field-annealed Co-based amorphous ribbons^32–34^, we recognize features in the MI curves that are compatible with the ones acquired for the biphase films. Specifically, we observe that the high-amplitude peak is located at a field whose orientation is parallel to the annealing field, here understood as the pre-magnetizing field (as we can see for instance in Fig. 3 of Ref.^32^, Figs. 2 and 3 in Ref.^33^ and Fig. 2 in Ref.^34^). In all these works, the results are interpreted in terms of an unidirectional magnetic anisotropy developed in the field annealing due to the direct exchange coupling between magnetic phases, inducing a bias field that favours the parallel alignment of the soft and hard phases. Remarkably, despite the difference between the nature of magnetic interactions present in films and ribbons, for both geometries the AMI results disclose fingerprints of ferromagnetic-like interlayer magnetic interactions.

For wires in turn, such as microwires constituted of a soft CoFe-based amorphous nucleus and a magnetically harder crystalline CoNi outer layer^29,30^, it is interesting to notice that the results are completely the opposite to the ones described so far. For this sample geometry, we notice from minor magnetization loops that the curves are shifted towards the orientation of the pre-magnetizing field and on the magnetization of the hard phase (Fig. 3 in Refs.^29,30^). It is consistent with the existence of the bias field of magnetostatic origin. This field arises from uncompensated poles at the ends of the pre-magnetized hard outer shell, and favours the antiparallel alignment of the soft and hard phases. As a consequence, the high-amplitude peak in MI curves is found at a field whose orientation is antiparallel to the pre-magnetizing field (as we can see in Fig. 5 of Ref.^30^ and Fig. 4 in Ref.^29^). Hence, AMI results in wires are ascribed to the magnetostatic interaction between the core and the shell, i.e. an antiferromagnetic-like interlayer coupling.

What lessons can be drawn from our work? In biphase ferromagnetic systems, asymmetric magnetoimpedance effect undoubtedly arises from the magnetic interaction between the soft and hard magnetic phases. The MI response of the system can be tuned by modifying the bias field acting on the soft phase, i.e. through the coupling of the soft phase with the hard one. The intensity of the bias field plays important role in the magnetic properties and dynamic magnetic behavior, influencing for instance the reversion field of the soft phase and the amplitude and peaks position of the MI response. Nevertheless, the nature of the interlayer magnetic interaction is the ingredient that plays the key role, settling the character of the coupling, the orientation of the bias field and, consequently, establishing the AMI profile.

By probing the quasi-static and dynamic magnetic properties of biphase ferromagnetic films, with soft and hard ferromagnetic phases intermediated by a non-magnetic layer, we have addressed aspects of the coupling between magnetic layers. Our results have undoubtedly demonstrated the ferromagnetic-like nature of the interlayer magnetic interactions in biphase films, revealing the main role of the Néel (orange-peel) coupling in such nanostructures. Our work helps to reinterpret the results of previous studies, raising the fundamental similarities and differences in the asymmetric magnetoimpedance of different systems. Further, it has consolidated our knowledge on biphase ferromagnetic films, a simple nanostructure that can be engineered to yield not only fundamentally interesting behaviors, but responses that have the potential to influence current and future technologies of auto-biased linear magnetic field sensors.

## Methods

### Set of samples

For our analysis, we reproduce Ni$_{81}$Fe$_{19}$(25 nm)/Cu($t_{\mathrm {Cu}}$)/Co(50 nm) films, with $t_{\mathrm {Cu}}= 5$, 7, and 10 nm, depositing the samples by magnetron sputtering onto glass substrates, with dimensions of $8 \times 4$ mm$^2$, following the procedures described in Ref.^39^.

### Magnetic characterization

For the magnetic characterization of our films, magnetization curves are acquired with a Lakeshore 7404 vibrating sample magnetometer, while the magnetoresistance measurements are performed with an experimental setup using the conventional four-probe method, in a way similar to that reported in Ref.^40^. At last, MI measurements are obtained over a wide range of frequencies, between 0.1 and 3 GHz, with 0 dBm (1 mW) constant power applied to the sample, using a RF-impedance analyzer Agilent model E4991, with a E4991*A* test head connected to a microstrip, following the procedures traditionally employed by our group^26,39^.

## Data Availability

All data generated or analysed during this study are included in this published article.
